# New Eco-Sustainable Feed in Aquaculture: Influence of Insect-Based Diets on the Content of Potentially Toxic Elements in the Experimental Model Zebrafish (*Danio rerio*)

**DOI:** 10.3390/molecules27030818

**Published:** 2022-01-26

**Authors:** Cristina Truzzi, Federico Girolametti, Leonardo Giovannini, Ike Olivotto, Matteo Zarantoniello, Giuseppe Scarponi, Anna Annibaldi, Silvia Illuminati

**Affiliations:** 1 Department of Life and Environmental Sciences, Università Politecnica delle Marche, Via Brecce Bianche, 60131 Ancona, Italy; i.olivotto@univpm.it (I.O.); m.zarantoniello@pm.univpm.it (M.Z.); g.scarponi@univpm.it (G.S.); a.annibaldi@univpm.it (A.A.); s.illuminati@univpm.it (S.I.); 2 Independent Researcher, 06065 Passignano sul Trasimeno, Italy; leonardo.giovannini@outlook.it; 3 Fano Marine Center, The Inter-Institute Center for Research on Marine Biodiversity, Resources and Biotechnologies (FMC), Viale Adriatico 1/N, 61032 Fano, Italy

**Keywords:** *Hermetia illucens*, cadmium, lead, arsenic, mercury, nickel, fish feed, aquaculture

## Abstract

According to the concept of circular economy, insects represent good candidates as aquafeed ingredients. Nevertheless, there are some potential chemical risks linked with insect consumption. In this study, we reared the teleost *Danio rerio*, used as an experimental model, with five experimental diets characterized by increasing levels (0%, 25%, 50%, 75%, and 100%) of full-fat *Hermetia illucens* (Hi) prepupae, substituting for fish meal (FM) and fish oil (FO). We investigated the presence of potentially toxic elements (PTEs) Cd, Pb, Ni, As, and Hg in larval (20 days), juvenile (2 months), and adult (6 months) fish. Quantitative determinations of Cd, Pb, Ni, and As were made with an atomic absorption spectrometer; the total mercury content was determined by a direct mercury analyzer. The substitution of FM and FO with *Hermetia illucens* meal led to a reduction in the content of some PTEs, such as Pb, As, and Ni, in fishfeed, leading to concentrations below the legal limit of undesirable substances in animal feed. By increasing the Hi meal dietary content, we observed in the *Danio rerio* specimens an increase in Cd, Pb, and Ni content and a reduction in As content for all life stages. Moreover, a general increase in the content of Cd, Pb, Hg, and Ni from larvae to juvenile was measured, while the shift of *Danio rerio* from the juvenile to the adult stage involved a significant increase in the content of Pb, Hg, and Ni. Larvae had a reduced ability to bioaccumulate metal(loid)s compared to juveniles and adults. In conclusion, the content of PTEs in *Danio rerio* is influenced both by the type of diet administered and by the life stage of the animal itself. This research demonstrates the possibility of using Hi prepupae as an aquafeed ingredient without exposing fish to a chemical risk and, in perspective, allows applying these eco-sustainable diets for the breeding of edible fish species, without endangering human health.

## 1. Introduction

On the basis of a forecast predicting a significant demographic increase of the global population (over nine billion people by 2050), the Food and Agriculture Organization of the United Nations [1] estimates that world food production will have to increase by 60% to meet food needs. Therefore, we are facing the challenge of searching for feed and food with an adequate nutritional profile that “conserves land, water, plant and animal genetic resources, and it is environmentally non-degrading, technologically appropriate, economically viable and socially acceptable” [2]. Insects are a solid way to produce green feed, to reduce food waste, and to fight climate changes [3,4]. In fact, insects are rich in proteins and lipids, show a low environmental footprint [5], and can convert low-quality carbohydrate-rich organic materials (such as food waste) into high-quality feed ingredients to be used in feed formulas [6,7], thereby supporting the circular economy and sustainable development. In aquaculture, insects have been proposed to substitute for fish meal (FM) and fish oil (FO), obtained from wild-caught fish, due to the severe depletion of fish resources [1,8,9]. However, regarding the chemical hazard associated with fish feed, both FM and insects may contain hazardous chemicals, including potentially toxic elements, PTEs. It is known in fact that FM is an important source of PTEs in fish feed [10,11,12,13,14]. Fish meal is made by drying and grinding the carcasses of fish, leading to a concentration of toxic elements present in the fish themselves. This fact, together with the contamination of marine environments, led to a product that contains excessive amounts of pollutants [12]. PTEs are naturally present in insects as well, but they can also be transferred to insects through the rearing substrate [15,16,17]. PTEs have a high degree of toxicity and a wide distribution in the environment [18,19,20,21,22]. Metals such as arsenic (As), nickel (Ni), cadmium (Cd), lead (Pb), and mercury (Hg) rank among the priority elements that are of great public health significance. The International Agency for Research on Cancer (IARC) in fact classifies Cd, As, and Ni as carcinogenic, and Pb and Hg as probably carcinogenic to humans [23]. Consuming foods containing PTEs can lead to numerous human health risks, such as renal disfunction and cancer (Cd) [24], neurological deficits (Pb) [25], dementia and dysarthria (Hg) [26], cardiovascular disorders and skin problems (As) [27], DNA damage, and immunologic and neurologic problems (Ni) [28].

PTEs can be accumulated in aquatic organisms through different routes, including respiration and ingestion [29,30]. Nowadays, there is a growing recognition that dietary metal(loid) exposure may contribute to chronic toxicity in aquatic organisms [11,31,32,33]. These chemical contaminants can be bioaccumulated in organisms or biomagnified in food chains [34,35]. Therefore, the consumption of fish contaminated with PTEs poses a serious risk of exposure to these toxic elements and leads to a potential risk for human health [36,37,38,39,40,41,42]. Given their dangerous nature, concerning feed and food safety and, ultimately, human health, these PTEs are monitored both in the environment [43,44] and in feed [45] and food [46,47,48]. Therefore, to achieve the safe production of insects to be used as feed in aquaculture, as well as investigate other substrates compatible with European rules regarding safety about chemical contaminants, a strict monitoring of PTEs content is necessary for all kind of feedstuff [49,50].

The *Hermetia illucens* (Hi, Diptera, *Stratiomydae*) has been proposed by the European Food Safety Authority Scientific Committee [3] as one of the species with the greatest potential as a food and feed ingredient in the European Union. It is characterized by a balanced essential amino acid profile, similar to that of FM, and represents a valuable protein source in fish diets [9,51,52,53]. Hi can bioaccumulate potentially toxic elements from the rearing substrate [15,16,17], but their content is generally lower than the legal limit for feed [45]. Therefore, Hi prepupae have been proposed as a substitute for FM in fish feed [54,55,56,57,58,59].

*Danio rerio* (zebrafish) is a perfect experimental model for scientific studies, because of its short life cycle, high reproductive rate, well-defined developmental processes, and abundant information on its genomic features, similar to that of vertebrates, including humans [60,61,62,63,64]. From an eco-toxicological perspective, zebrafish have been extensively used to study heavy metals, endocrine-disrupting chemicals, and persistent organic pollutants [65,66,67,68,69]. However, in most studies, high amounts of PTEs [69,70,71,72,73,74,75], which bear no relation to their content in the environment, were added to the fish feed or the aquatic environment, whereas studies wherein “natural” contaminants content were determined in zebrafish at various life stages are scarce [32,33,76].

This study is part of the research project NUTRIFISH (Cariverona fundation, code n. 2017.0571), aimed at studying the possibility of including *Hermetia illucens* (Hi) meal, grown on substrates suitably enriched with polyunsaturated fatty acids (silverskin, coffee-roasting residue, and microalgae) in aquafeeds intended for aquaculture. In the present study, full-fat Hi prepupae meal was obtained from Hi larvae reared on a coffee by-product (coffee silverskin) enriched with 10% of microalgae *Schizochytrium* sp. [77]. The aim of this study is to evaluate the chemical risk associated with the use of Hi-based diets on PTEs content using zebrafish as an experimental model. In accordance with European directives [44,45], we evaluated the presence of Cd, Pb, As, Ni, and Hg, in *Danio rerio* fed insect-based experimental diets from larvae to adults of zebrafish, in order to evaluate possible accumulation during the whole life cycle of the fish and to assess the possibility of applying these diets to commercial fish products normally used in human diet.

## 2. Results

### 2.1. Potentially Toxic Elements in Water Tanks

PTEs’ total concentrations are reported in Table 1. Hg and Pb levels showed values below the instrumental LOD (0.6 and 30 ng L^−1^, respectively). As for other PTEs, the measured content was, from the lowest to the highest: Cd < As < Ni. In general, the content of PTEs were well below the legal limits set by Italian Legislative Decree 31/2001 [43], which regulates the quality of water intended for human consumption in order to protect human health from the negative effects deriving from water contamination.

### 2.2. Potentially Toxic Elements in the Experimental Diets

The PTEs content of diets used in the experimental design is shown in Table 2, as mg kg^−1^ ww for a moisture level (M) of 12%, for a comparison with legal limits of Directive 2002/32/EC [45] on undesirable substances in animal feed. PTEs content in Hi meal is also reported in Table 2 (from [16]) in support of the discussion of the results.

Cd content varied from 0.31 to 0.51 mg kg^−1^ ww (12% M) (Table 2), corresponding to a range from 0.35 to 0.58 mg kg^−1^ dw, of the same order of magnitude as the content of Cd in the Hi prepupae used to prepare the insect meal (0.20 ± 0.02 mg kg^−1^ ww, 12% M). A statistically significant decrease of Cd content was evidenced (*p* = 0.0022) in the Hi25 diet with respect to the Control diet, whereas the major inclusion of Hi meal substituting for FM led to a statistically significant increase of Cd content (*p* = 0.0022) in Hi50, Hi75, and Hi100 diets with respect to the Control diet and the Hi25 diet. Referring to the EC limit [45] on undesirable substances in animal feed, the mean value of Cd content, 0.42 ± 0.08 mg kg^−1^ ww (12% M), was lower than the legal limit for fish feed.

Pb content varied from 0.31 to 0.71 mg kg^−1^ ww (12% M) (Table 2), corresponding to a range from 0.35 to 0.81 mg kg^−1^ dw, about an order of magnitude greater than the content of Pb in the Hi prepupae used to prepare the insect meal, equal to 0.059 ± 0.007 mg kg^−1^ ww (12% M). With the increase in the percentage of HI meal in the experimental diets, a statistically significant decrease of Pb content was evidenced (*p* < 0.0001); in particular, Hi75 and Hi100 diets showed a Pb content significantly lower than the Hi50 diet, which in turn showed a significantly lower Pb content with respect to the Hi25 and Control diets. Referring to the EC limit [45] on undesirable substances in animal feed, the mean value of Pb content, 0.48 ± 0.18 mg kg^−1^ ww (12% M), was about ten-fold lower than the legal limit of 5 mg kg^−1^ ww both for animal feed and fish feed.

Hg content varied from 0.032 to 0.037 mg kg^−1^ ww (12% M) (Table 2), corresponding to a range from 0.036 to 0.042 mg kg^−1^ dw. These values are of the same order of magnitude as the Hg content in Hi prepupae used to prepare insect meal (0.053 ± 0.03 mg kg^−1^ ww, 12% M). No statistically significant differences were evidenced between tested diets (*p* > 0.05). Referring to the EC limit [45] on undesirable substances in animal feed, the mean value of Hg content, 0.034 ± 0.002 mg kg^−1^ ww (12% M), was about six-fold lower than the legal limit of 0.2 mg kg^−1^ ww reported for complete feed for fish.

Arsenic content varied from 0.11 to 0.21 mg kg^−1^ ww (12% M) (Table 2), corresponding to a range from 0.13 to 0.24 mg kg^−1^ dw. The Control diet showed an Arsenic content about 2-fold higher than the As content in Hi meal (0.109 ± 0.003 mg kg^−1^ ww, 12% M). Consequently, with the increase in the percentage of Hi meal in the diets from 0% (CTRL) to 25% (Hi25) and to 50% (Hi50), a statistically significant decrease of As content was evidenced (*p* < 0.0001). No statistically significant differences were evidenced in As content among Hi50, Hi75, and Hi100 diets. Referring to the EC limit [45] on undesirable substances in animal feed, the mean value of As content, 0.14 ± 0.04 mg kg^−1^ ww (12% M), was about 70-fold lower than the legal limit of 10 mg kg^−1^ ww reported for complete feed for fish.

Ni content varied from 0.11 to 1.72 mg kg^−1^ ww (12% M) (Table 2), corresponding to a range from 0.12 to 1.95 mg kg^−1^ dw. The Ni content of the Control diet is greater than the content of Ni in the Hi prepupae used to prepare insect meal, 1.06 ± 0.04 mg kg^−1^ ww (12% M). In parallel to the increase in the percentage of Hi meal in the diets, a statistically significant decrease of Ni content was evidenced (*p* < 0.0001), with a reduction of about 15 times from the Control diet to the Hi100 diet.

**Table 2:** PTEs content (mg kg^−1^ ww) in Hi meal and feed, referring to the EC limits [45] on undesirable substances in complete feed (maximum content relative to a feeding stuff with a moisture content of 12%).

Diets	Cd	Pb	Hg	As	Ni
Legal Limit for Fish Feed	1.0	5.0	0.2	10	-
Hi meal *	0.20 ± 0.02	0.059 ± 0.007	0.053 ± 0.003	0.109 ± 0.003	1.06 ± 0.04
CTRL	0.38 ± 0.02 ^b^	0.63 ± 0.04 ^c^	0.033 ± 0.001 ^a^	0.21 ± 0.01 ^c^	1.72 ± 0.04 ^d^
Hi25	0.31 ± 0.02 ^a^	0.71 ± 0.04 ^c^	0.035 ± 0.001 ^a^	0.14 ± 0.01 ^b^	1.04 ± 0.11 ^c^
Hi50	0.45 ± 0.02 ^c^	0.42 ± 0.02 ^b^	0.037 ± 0.001 ^a^	0.12 ± 0.01 ^a^	0.20 ± 0.02 ^b^
Hi75	0.51 ± 0.03 ^c^	0.34 ± 0.02 ^a^	0.032 ± 0.001 ^a^	0.12 ± 0.01^a^	0.21 ± 0.03 ^b^
Hi100	0.46 ± 0.04 ^c^	0.31 ± 0.02 ^a^	0.034 ± 0.002 ^a^	0.11 ± 0.01 ^a^	0.11 ± 0.01 ^a^

Diets Hi25, Hi50, Hi75, and Hi100: increasing inclusion levels (25%, 50%, 75%, and 100% respectively) of full-fat *Hermetia illucens* prepupae in substitution of fish meal and fish oil referred to the Control diet (CTRL). * From Truzzi et al. [16]. Means within columns bearing different letters are significantly different (*p* < 0.05).

### 2.3. Potentially Toxic Elements in Danio rerio

**Figure 1:**
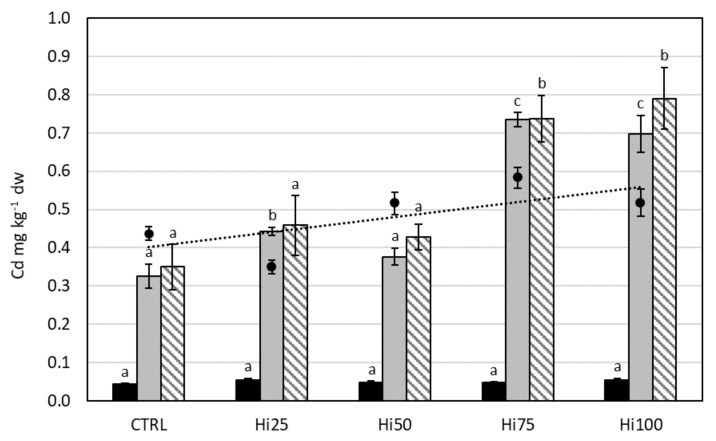
Cadmium content (mg kg^−1^ dw) in diets (dark spot, dashed trend line) and in larvae (black bars), juveniles (gray bars), and adults (striped bars) of *Danio rerio* fed with experimental diets: CTRL, control; Hi25, Hi50, Hi75, and Hi100, diets enriched with 25, 50, 75, and 100% of Hi full-fat prepupae, respectively. The values are presented as mean ± standard deviation (*n* = 3). Different letters indicate statistically significant differences between specimens of the same life stage (*p* < 0.05).

**Figure 2:**
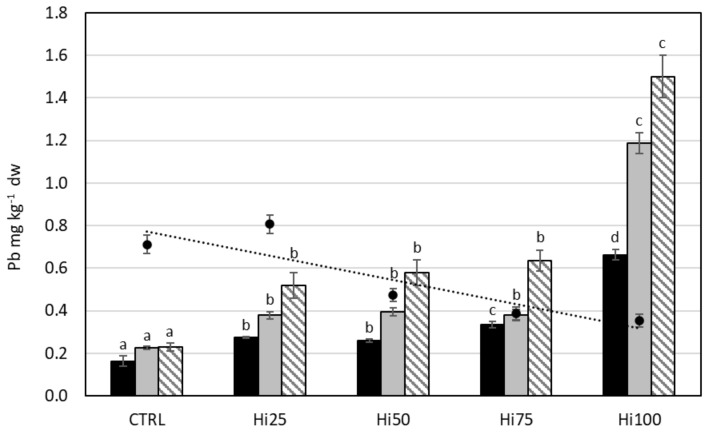
Lead content (mg kg^−1^ dw) in diets (dark spot, dashed trend line) and in larvae (black bars), juveniles (gray bars), and adults (striped bars) fed with experimental diets: CTRL, control; Hi25, Hi50, Hi75, and Hi100, diets enriched with 25, 50, 75, and 100% of Hi full-fat prepupae, respectively. The values are presented as mean ± standard deviation (*n* = 3). Different letters indicate statistically significant differences between specimens of the same life stage (*p* < 0.05).

**Figure 3:**
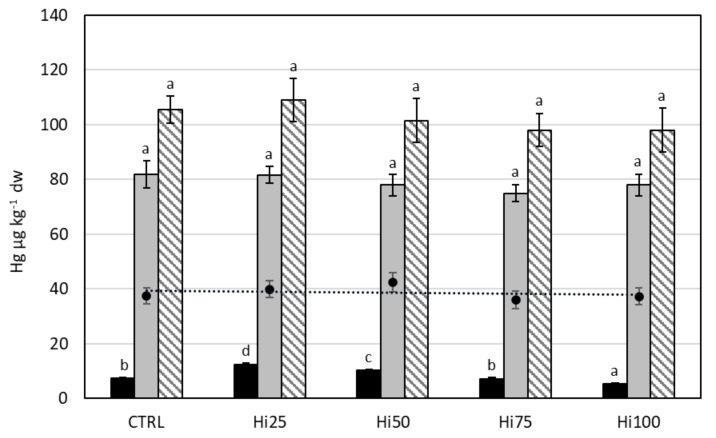
Mercury content (µg kg^−1^ dw) in diets (dark spot, dashed trend line) and in larvae (black bars), juveniles (gray bars), and adults (striped bars) of *Danio rerio* fed with experimental diets: CTRL, control; Hi25, Hi50, Hi75, and Hi100, diets enriched with 25, 50, 75, and 100% of Hi full-fat prepupae, respectively. The values are presented as mean ± standard deviation (*n* = 3). Different letters indicate statistically significant differences between specimens of the same life stage (*p* < 0.05).

**Figure 4:**
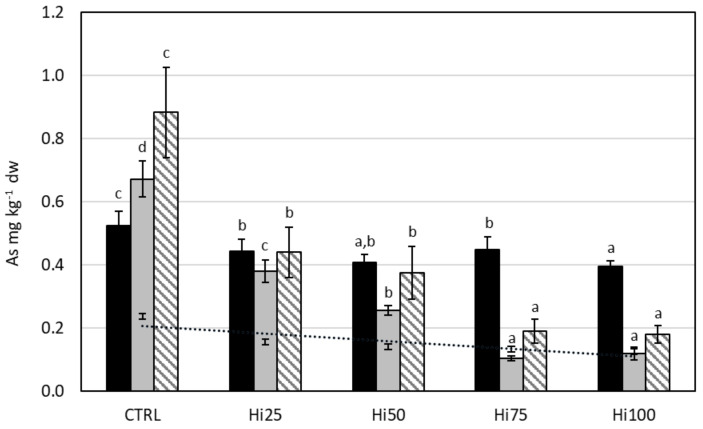
Arsenic content (mg kg^−1^ dw) in diets (dark spot, dashed trend line) and in larvae (black bars), juveniles (gray bars), and adults (striped bars) of *Danio rerio* fed with experimental diets: CTRL, control; Hi25, Hi50, Hi75, and Hi100, diets enriched with 25, 50, 75, and 100% of Hi full-fat prepupae, respectively. The values are presented as mean ± standard deviation (*n* = 3). Different letters indicate statistically significant differences between specimens of the same life stage (*p* < 0.05).

**Figure 5:**
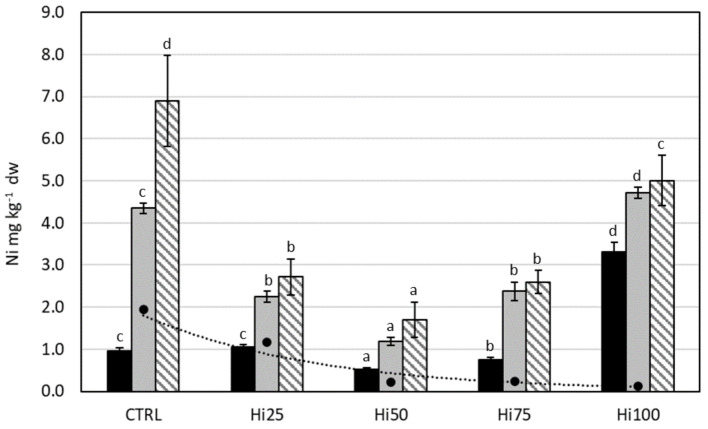
Nikel content (mg kg^−1^ dw) in diets (dark spot, dashed trend line) and in larvae (black bars), juveniles (gray bars), and adults (striped bars) of *Danio rerio* fed with experimental diets: CTRL, control; Hi25, Hi50, Hi75, and Hi100, diets enriched with 25, 50, 75, and 100% of Hi full-fat prepupae, respectively. The values are presented as mean ± standard deviation (*n* = 3). Different letters indicate statistically significant differences between specimens of the same life stage (*p* < 0.05).

PTEs content of zebrafish specimens (larvae, juvenile and adult) is reported in Figure 1, Figure 2, Figure 3, Figure 4 and Figure 5 (mg kg^−1^ dw). Moreover, PTEs content in experimental diets is also reported in Figure 1, Figure 2, Figure 3, Figure 4 and Figure 5 (mg kg^−1^ dw), for a visual comparison. Table 3 shows the bioaccumulation factor (BAF) for zebrafish in the investigated life stages, calculated on a dry-weight basis.

#### 2.3.1. Cadmium

Zebrafish larvae showed Cd content from 0.044 to 0.054 mg kg^−1^ dw (from 0.0068 to 0.0082 mg kg^−^1 ww), and no significant differences were evidenced among groups (*p* > 0.05) (Figure 1). All groups of larvae had a statistically significant lower Cd content than juvenile and adult specimens (*p* < 0.001). Juvenile and adult specimens fed with the same diet showed a similar Cd content, which ranged from 0.32 to 0.73 mg kg^−1^ dw (0.08–0.16 mg kg^−1^ ww) and from 0.35 to 0.79 mg kg^−1^ dw (0.11–0.25 mg kg^−1^ ww), respectively. Moreover, Cd concentration in juvenile and adult zebrafish fed with Hi75 and Hi100 diets increased significantly compared to specimens fed with the diets including lower percentages of Hi meal (*p* < 0.01). The BAF for larvae is about 0.1 and in juvenile and in adult specimens is about 1 (Table 3), apart from specimens fed with Hi75 and Hi100 diet, which showed a BAF of just over 1.

#### 2.3.2. Lead

Within the same life stage, the Pb content in zebrafish specimens increased significantly as the percentage of insect inclusion in the diet increased (*p* < 0.0001) (Figure 2): from 0.16 (CTRL) to 0.66 (Hi100) mg kg^−1^ dw in larvae, from 0.23 (CTRL) to 1.19 (Hi100) mg kg^−1^ dw in juveniles, and from 0.23 (CTRL) to 1.30 (Hi100) mg kg^−1^ dw in adults. By consequence, for each life stage, zebrafish specimens fed with insect-based diets showed a significantly higher Pb content than specimens fed with the control diet. In addition, fish fed with the Hi100 diet showed a significantly higher Pb content than fish of the same life stage fed with the Hi25, Hi50, and Hi75 diets, which generally showed no statistically significant differences between them. Comparing the different life stages, we observed how, in the fishes fed with the same diet, the content of lead increased significantly, passing from the larvae to the juveniles (*p* < 0.05) and from the juveniles to the adults (*p* < 0.05), except in the case of juveniles and adults fed with the Control diet, which did not show a statistically significant difference between them. In general, for each life stage, BAF increased as the percentage of insect meal included in the diet increased, reaching a value >1 in specimens fed with the Hi100 diet and for adults fed with the Hi75 diet (Table 3).

#### 2.3.3. Mercury

Figure 3 shows the content of mercury in the *Danio rerio* specimens in the various phases of the life cycle (larvae, juveniles, and adults). Larvae showed a statistically significant reduction of Hg content with increasing percentage of the inclusion of Hi meal in the diet (*p* < 0.0001), passing from 12.4 µg kg^−1^ dw for larvae fed with the Hi25 diet to 5.5 µg kg^−1^ dw for larvae fed with the Hi100 diet. Juveniles showed a Hg content of ~80 µg kg^−1^ dw, and no statistically significant differences between specimens bred with different diets (*p* > 0.05) were shown. The same result has been obtained for adult specimens, since all groups showed a Hg content of ~100 µg kg^−1^ dw. Comparing the different life stages between fishes fed with the same diet, the concentration of Hg increased significantly (*p* < 0.01) passing from the larvae to the juveniles and, finally, to the adult specimens. BAF values (0.1–0.3 for larvae specimens, ~2 for juveniles, and ~2.7 for adults) highlighted a different bioaccumulation capacity among the life stages considered.

#### 2.3.4. Arsenic

For each life stage, all groups of specimens fed with insect-based diets showed a statistically lower As content than specimens fed with the Control diet (*p* < 0.0001) (Figure 4). Moreover, zebrafish larvae fed with the Hi100 diet showed a significant decrease (*p* < 0.05) in As content (0.39 mg kg^−1^ dw) compared to larvae fed with the Hi25 and Hi75 diets (0.44 and 0.45 mg kg^−1^ dw, respectively) (Figure 4), but the decrease is much less pronounced than the decrease shown in juveniles and adults. In particular, as the percentage of insect inclusion in the diet increases, the As content decreased significantly (*p* < 0.0001) passing: (i) in juveniles, from 0.38 mg kg^−1^ dw in specimens fed with Hi25 diet, to 0.12 mg kg^−1^ dw in fish fed with the Hi100 diet; (ii) in adult, from 0.44 mg kg^−1^ dw in specimens fed with Hi25 diet to 0.18 mg kg^−1^ dw in fish fed with the Hi100 diet. As for a comparison among life stages, in fish fed with the control diet, the content of As increased significantly from larvae to juveniles and, finally, to adults (*p* < 0.05). In specimens fed diets containing Hi meal, in contrast, larvae had an equal or higher As content than juveniles and adults. The BAF for arsenic is generally >1 for all groups of considered life stages (larvae, 2–3.4; juveniles, 1.8–2.8; adults, 1.4–3.7), except for juveniles fed with Hi75 (0.8) and Hi100 (0.9) diets.

#### 2.3.5. Nickel

The trend of Ni content in zebrafish as a function of the administered diets was different from other studied PTEs and was similar for the different life stages (Figure 5). In particular, with the increase in the percentage of Hi meal in the diet, there was a significant decrease from specimens fed with the control diet to those fed with the insect-based diets Hi25 and Hi50. Then, a further increase in the percentage of Hi meal in the diet from 75% to 100% led to a statistically significant increase of Ni content in zebrafish specimens (*p* < 0.0001 for larvae, juveniles, and adults). The significantly lowest Ni content has been found in individuals fed the Hi50 diet for all considered life stages. As for the comparison among life stages, we observed how, in specimens fed with the same diet, the content of Ni increased significantly passing from larvae to juveniles (*p* < 0.05) for all considered diets and from juveniles to adults (*p* < 0.05) for specimens fed with Control, Hi25, and Hi50 diets. Concerning the BAF, all specimens considered showed a value >1, except for larvae fed with the Control and Hi25 diets. For all life stages, we observed an exponential growth of BAF with increasing percentage of the inclusion of Hi meal in the diet, from specimens fed the Hi25 diet to specimens fed the Hi100 diet.

## 3. Discussion

### 3.1. Potentially Toxic Elements in the Experimental Diets

Several studies have confirmed that food can be a major source of trace metals in fish [32,33,78,79], and fish feed is the major food for farmed fish. Therefore, it is important to find substitutes of FM that can reduce the content of these toxic elements in the diet. In turn, the content of PTEs in insects depends on the rearing substrate used for their growth [15,16,17]. Hi prepupae used in this study were growth with a substrate based on by-products obtained from roasting coffee (coffee silverskin) added with 10% (*w*/*w*) of *Schizochytrium* sp. [58]: the study demonstrated that Hi prepupae accumulate Cd, Pb, and Hg in their body; however, the content of PTEs was lower than the legal limit for feed (data reported in Table 3).

The results of this study concerning the content of PTEs in the diets administered to *Danio rerio* allow us to make the following considerations.

Cd and Hg concentrations found in experimental diets (mean, 0.47 ± 0.09, and 0.038 ± 0.002 mg kg^−1^ ww, respectively) of this study are of the same order of magnitude of their content in complete Atlantic salmon feed used from 2008 to 2017, 0.2–0.3 mg kg^−1^ ww for Cd and 0.050 ± 0.0070 mg kg^−1^ ww for Hg [10] or in complete feed used for *Seriola dumerili* (0.41-0.60 mg kg^−1^ ww for Cd), wherein FM has been replaced with alternative protein sources, such as corn gluten meal, krill meal, and meat and bone meal [80]. The concentration of arsenic (mean, 0.16 ± 0.05 mg kg^−1^ ww) was one order of magnitude lower than the complete Atlantic salmon feed in 2008–2017, 2.6 mg kg^−1^ ww [10] and in complete feed for *Seriola dumerili*, 1.86–3.15 mg kg^−1^ ww [80]. The Pb content of the experimental diets (mean, 0.54 ± 0.2 mg kg^−1^ ww) was one order of magnitude greater with respect to complete feed for Atlantic salmon from 2008 to 2017, 0.05–0.07 mg kg^−1^ ww [10]. The inclusion of Hi meal in substitution of FM and FO modified the content of some PTEs in the experimental diets. In particular, the increase of the inclusion level of Hi prepupae meal in the diets in substitution of FM resulted in a statistically significant (*p* = 0.0022) decrease of Pb, As, and Ni, whereas Hg content did not show significant changes. Moreover, a statistically significant decrease of Cd content was evidenced (*p* = 0.0022) in Hi25 with respect to the Control diet, whereas the further inclusion of Hi meal in substitution of FM led to a statistically significant increase of Cd content (*p* = 0.0022) in Hi50, Hi75, and Hi100 diets with respect to the Control diet and the Hi25 diet. These results are in line with the fact that, compared to FM and FO, Hi meal showed a similar Cd and Hg content, and a lower Pb, As, and Ni content. In consequence, the substitution of FM and FO with Hi meal determined a change in PTEs content in feed. These results agreed with literature data. Biancarosa et al. [57] demonstrated that the increased dietary inclusion of Hi meal in substitution of FM and FO in feed provided to Atlantic salmon *Salmo salar* led to an increase in the Cd content, whereas Hg did not show statistically significant differences between the tested feed. For Pb, contrary to what we found, they showed a slight increase with the increase of Hi meal in the diet. Monge-Ortiz et al. [80] found that FM substitution with alternative protein sources led to a decrease of As content in feed, and Hg did not show significant changes, as observed in the experimental diets of this study. However, Cd content showed a decrease passing from a diet based on FM to a diet wherein FM were replaced with corn gluten meal, krill meal, and meat and bone meal. The discrepancy of the results is probably tied to the different kind of fish feed used for the fish farm.

All investigated PTEs showed a lower content than legal limits intended for feed [45] (except for the Ni, for which no legal limit is reported in the regulation in force); therefore, there is no chemical risk associated with the presence of these elements. This result made it possible to apply the same type of experimental diet to a species of edible fish, the sturgeon [81], as part of the NUTRIFISH Project. In the immediate future, this finding opens the possibility of exploiting an eco-sustainable resource, such as insects, for the breeding of edible species in aquaculture, reducing the exploitation of fish species to produce FM and FO.

### 3.2. Potentially Toxic Elements in Danio rerio

The PTEs content in fish is a function of their concentration in the environment and in the diet and of the exposure period [82]. PTEs levels in the diets (min–max, mg kg^−1^ dw: Cd 0.35–0.58, Pb 0.35–0.81, As 0.13–0.24, Ni 0.12–1.95, Hg 0.036–0.042) showed a content much higher with respect to PTEs concentration in tank water (Table 3): about 5 orders of magnitude for Cd and Pb, 4 for As and Hg, and 2–3 for Ni. These results suggested that the PTEs content in water is negligible; therefore, we can assume that the PTEs content in zebrafish specimens was influenced almost exclusively by the PTEs content in the diet.

#### 3.2.1. Cadmium

The mean value of Cd content for the different life stages was 0.050 ± 0.04, 0.5 ± 0.2, 0.5 ± 0.2 mg kg^−1^ dw for larvae, juvenile, and adults, respectively. These results were consistent with literature data (Table 4) for zebrafish specimens subjected to experiments in aquaria [32,33,69,70,71,75], within the limits of the experimental error. In particular, Cd content in larvae (0.007 mg kg^−1^ ww) was similar to Cd content in larvae from Boyle [32], ~0.02 mg kg^−1^ ww; adult specimens showed a Cd content (0.35–0.79 mg kg^−1^ dw, 0.11–0.25 mg kg^−1^ ww) similar to the Cd content in adult zebrafish from Lacave et al. [69], 1.5 mg kg^−1^ dw, from Gonzalez et al. [75], 0.200 ± 0.03 mg kg^−1^ dw, and from Zhan et al. [83], ~0.75 mg kg^−1^ ww. The obtained results showed that the concentration of this metal increases both with the growth of zebrafish (and then with the exposure time) and with the increase of the percentage of insect meal present in the feed. In fact, juveniles and adults have a content of Cd about 10 times greater than larvae; in addition, juveniles and adults fed on the highest insect meal inclusion diets (Hi75 and Hi100) showed a statistically higher Cd content than other groups. In literature increasing evidence has shown that several factors influence the accumulation of Cd in fish tissues, such as the concentration of this metal in the environment or in the diet and the exposure time [68,71,84,85]. The BAF for larvae is about 0.1, and in juvenile and adult specimens, it is about 1, emphasizing how Cd accumulation depends also on the life stage. A bioaccumulation factor ≤1 indicates no bioaccumulation; therefore, the Cd reaches its highest concentration in the juveniles and then remains constant in the adult specimens, without bioaccumulation. Previous literature demonstrated that Cd is accumulated by different organs due to different doses of Cd^2+^ treatment and different exposure times [82,86]: in these cases, differently from our study, higher levels of Cd were added in the tank water. In addition, aquatic Cd binds generally more efficiently in fish than dietary Cd [87], and we demonstrated a very low Cd content in the aquatic media of the zebrafish farm (Table 1). Our data were consistent with data reported by Rehwoldt and Karimian-Teherani [88], which showed an increase in Cd concentration in the zebrafish exposed to 10 ppm of Cd added in feed, related to the exposure time: Cd content reached a plateau of about 5 mg kg^−1^ dw after 2–3 months of exposure. In our study, the juvenile specimens of 2 months had the same Cd content of the adult specimens, and an equilibrium was reached between the rate of retention and the rate of excretion. It is known that, in fish, low levels of Cd induce metallothionein formation [89,90]; thus the low accumulation of the metal in the fish tissues is possibly due to the binding of the metal in the liver with metallothionein molecules with its subsequent depuration from the organs. We can therefore conclude that the Cd present in the feeding substrate enter the body of zebrafish in different life stages, but it is excreted without bioaccumulation.

#### 3.2.2. Lead

The mean value of Pb content for the different life stages was 0.3 ± 0.2, 0.5 ± 0.2, 0.6 ± 0.4 mg kg^−1^ dw for larvae, juvenile, and adults, respectively. It is not possible to compare our results with literature data, since usually zebrafish specimens have been exposed to elevated concentrations of Pb in the water tank for toxicological studies, and Pb content was measured in different tissues [68,83]. Although the content of Pb in the experimental diets tends to decrease as the percentage of Hi meal increases, zebrafish specimens of all life stages showed an increase in Pb concentration, passing from the Control diet to the Hi100 diet. These results could appear to contrast with studies conducted on zebrafish exposed to contaminated river water [68] or to high Pb concentration in tank water [83], which showed that lead tends to bioaccumulate in the body, especially in gills and liver, as the external concentration of Pb increases, reaching a concentration of 0.2–38 mg kg^−1^ ww in gills and liver for exposure to ~16 mg·L^−1^ [68], or of about 2 mg kg^−1^ ww in the carcass for exposure to 1 ppm [83]. But it is known that Pb accumulation depends on several factors, including developmental stage, water quality, Pb concentration, and variability within and among populations and/or species [30,91]. Moreover, aquatic organisms take up lead from both the surrounding water and the diet, but it was demonstrated that the effects of dietary exposure to lead are relatively minor [92]. From the results of this study, we can hypothesize that the Pb present in Hi meal, although it is present in much lower concentrations than in the Control diet, is present in a more bioavailable and more easily assimilated form by zebrafish specimens in the various life stages considered. This hypothesis is supported by the value of the bioaccumulation factor found in zebrafish specimens: within each life stage, the BAF increases as the percentage of the inclusion of insect meal in the diet increases, highlighting the ability of fish to bioaccumulate Pb only in animals fed with diets containing the highest percentages of insect meal (Hi100), for larvae, juvenile, and adults (BAF 1.9 ± 0.1, 3.4 ± 0.1, 3.7 ± 0.4 respectively). These results agree with literature studies, which demonstrated that lead has little capacity to accumulate in fish muscle [14,93,94], while liver, kidney, and scales are the main sites for Pb accumulation [90,95], and similarly for zebrafish [68,83]. In addition, within the same diet administered, there is an increase in the content of Pb passing from larvae to juveniles and to adult specimens. The ability of zebrafish to uptake Pb could therefore depend both on the bioavailability of Pb present in the diet and on the life stage of the animal itself.

#### 3.2.3. Mercury

The mean value of Hg content for the different life stages was 0.009 ± 0.003, 0.079 ± 0.003, 0.102 ± 0.005 mg kg^−1^ dw for larvae, juvenile, and adults, respectively. These results, within the limits of the experimental error, were consistent with literature data (Table 4), recorded in adult specimens of zebrafish, wherein Hg content was next to 0 mg kg^−1^ dw [96]. It is known that the Hg content in fish depends on many factors, such as the presence in the environment [11] and in the diet [70] and the trophic level of the species [29,97,98]. Moreover, Hg distribution in fish depends on the life stage and on the Hg’s affinity for different tissues. In zebrafish larvae, Hg is highly localized in the rapidly dividing lens epithelium, with lower levels going to brain, optic nerve, and various other organs [99], whereas adult zebrafish specimens fed with Hg-enriched diets accumulate the metal in muscle, liver, and brain, dependent on the Hg content in the diet and on the exposure time [68,70,100].

In this study, larvae showed a significant reduction in Hg content as the percentage of Hi meal inclusion in the diet increases, thus decreasing from Hi25-fed to Hi100-fed individuals (*p* < 0.01). For both juvenile and adult specimens, the Hg content did not differ significantly between groups (*p* > 0.05). Zarantoniello et al. [78] demonstrated that higher inclusion levels of Hi meal in the diets (i.e., Hi75 and Hi100) affect larval liver histology of zebrafish and induce a general increase in lipid accumulation, unlike juveniles, who showed a decrease in total lipid content in specimens fed with the Hi75 and Hi100 diets with respect to the other experimental groups of the same life stages [101], and unlike adults, who showed a similar lipid content among groups (data not published). Several studies demonstrated a negative correlation between lipid content and Hg accumulation in fish [41,102]. Moreover, high inclusion levels of Hi meal in the diet lead to an alteration of molecular markers related to the appetite stimulus [78]. These physiological alterations could contribute to change the accumulation capacity of larvae towards mercury.

The Hg content increased as a function of the growth of the fish being studied: from larvae to juveniles and from juveniles to adults (*p* < 0.0001), reaching in adults a concentration of Hg about 10 times higher with respect to larvae. These results confirm the dependence of Hg content in fish on the exposure time, demonstrated by Gonzalez et al. [96]: zebrafish fed with 5 µg of Hg g^−1^ dw contaminated food showed an increase in Hg content in muscle from day 7 (~3 mg kg^−1^ dw) to day 63 (~15 mg kg^−1^ dw) [72]. Larvae, which showed a statistically significant lower Hg content with respect to juveniles and adults, were not able to bioaccumulate Hg (BAF equal to about 0.2). Increasing the exposure time, Hg content in zebrafish specimens increased, then juveniles (BAF about 2) and even more adults (BAF about 2.7) bioaccumulated this metal. This data confirmed the bioaccumulation capacity of Hg in living organisms, as reported in the literature [29,30,98].

#### 3.2.4. Arsenic

The mean value of As content for the different life stages was 0.43 ± 0.03, 0.3 ± 0.2, 0.4 ± 0.3 mg kg^−1^ dw for larvae, juveniles, and adults, respectively. These results are consistent with literature data (Table 4) for adult zebrafish specimens, within the limits of the experimental error. In particular, adult specimens showed a content of As (0.18–0.88 mg kg^−1^ dw) similar to As content in adult zebrafish from Wang et al. [76], 0.94 ± 0.08 mg kg^−1^ dw, and from Boyle et al. [33], 0.27–0.39 mg kg^−1^ dw. Larvae exposed to 5 ppm of As in a water tank showed As content about ten-fold higher (0.4–0.5 mg kg^−1^ ww) than larvae from this study [73].

For arsenic, results showed that the increase of the inclusion level of Hi prepupae meal in the diets, which corresponds to a decrease in As in feed, is accompanied by a reduction in the content of this toxic element in zebrafish, especially in juvenile and in adult specimens, and less markedly in larvae. The higher (or similar) content of As in larvae fed with Hi meal-based diets with respect to juveniles and adults could be explained by the lower larval metabolism, which slows down detoxification processes (such as methylation capacity) and the excretion of toxic substances [103]. Our results demonstrated a statistically significant linear positive relationship between As content in the diets and As content in the different zebrafish life stages: larvae (*p* = 0.0272, r = 0.9193), juvenile (*p* = 0.0090, r = 0.9616), and adult (*p* = 0.0036, r = 0.9792); therefore, we can deduce that As content in zebrafish is influenced by the level of As in the diet. This statement is supported by literature data: some authors demonstrated that dietary uptake could be the primary route for As bioaccumulation in fish [104,105], and a correlation between As content in the diet and As content in fish tissues has been already demonstrated in *Danio rerio* [106,107].

From the analysis of the BAF for As (2.0–3.4 for larvae; 0.9–2.8 for juvenile; 1.4–3.7 for adult), an ability was observed in zebrafish to bioaccumulate such metalloids, as demonstrated in the literature [33,68,73]. In particular, adult zebrafish exposed to contaminated river waters in an aquarium under static conditions for 7 consecutive days showed a BAF of 0.45 in gills and of 2.26 in liver [68]. Boyle [33] demonstrated the accumulation of As in adult zebrafish fed with the polychaete *Nereis diversicolor* collected from a metal-impacted estuary. Several studies demonstrated the ability of other fish species to accumulate As, such as striped snakehead (*Channa striata*) [108], juvenile rockfish (*Sebastes schlegelii*) [109], Tilapia (*Oreochromis spp*) [110], or brown trout (*Salmo trutta*) [111]. Bioaccumulation already occurs in the larval stage as the arsenic is absorbed through aquaglyceroporins (membrane proteins similar to mammalian aquaporins) that develop in the early stages of the life of the fish, so much so as to determine concentrations in larvae equal to those in the muscles of adults, as demonstrated by Hamdi et al. [73]. In that study, after a short-term exposure (2–6 days post fertilization for larvae, 96 h for adult) to 5 ppm sodium arsenite, an As concentration of about 0.5 mg kg^−1^ ww was recorded in larvae and in the muscle of adult specimens.

Arsenic is an element of concern for feed and food safety. Being naturally present in pelagic fish, it is transferred to marine feed ingredients, i.e., FM and FO, which are the major sources of arsenic in aquafeeds [14]. Due to the influence of the diet on As content in fish, the substitution of FM with an insect-based diet has the advantage of reducing the level of this metalloid in fish tissues.

#### 3.2.5. Nickel

The mean value of Ni content for the different life stages was 1.3 ± 0.03, 3 ± 1, 4 ± 2 mg kg^−1^ dw for larvae, juveniles, and adults, respectively. These results were consistent (Table 4) with literature data for zebrafish, within the limits of the experimental error. Alsop et al. [74] recorded in the carcass of zebrafish a mean value of As content of 2.19 ± 0.19 mg kg^−1^ ww, a value similar to that found in this study (0.54–2.1 mg kg^−1^ ww). 

Ni content in zebrafish specimens did not seem to be correlated to the Ni level in diets, because, as the Ni content in the insect-based diets decreases, a general increase in the Ni content in the fish was observed. In line with this result, Alsop et al. [74] also demonstrated that there were no differences in tissue Ni concentrations in zebrafish fed with a control diet or a Ni-enriched diet. To explain the obtained results, the increased bioavailability of Ni in insect-based diets compared to the control diet could be called into question, as previously proposed for lead. However, the existence has been demonstrated in zebrafish of several very complex stimulatory and inhibitory interactions among metals, such as Cd, Pb, Cu, Zn, and Ni, during the uptake process [112]. Therefore, the apparent strange behavior of Ni could be due to different causes, for which this study did not provide enough information, and further investigation is needed.

Concerning the BAF, all the considered specimens showed a value >1, except for larvae fed with the Control diet and the Hi25 diet. The bioaccumulation capacity is greater in juveniles (BAF 2.2–39) and adults (BAF 3.5–42) than in larvae (0.49–27). For all life stages, we observed an exponential growth of BAF with an increasing percentage of the inclusion of Hi meal in the diet, from the specimens fed with Hi25 to those fed with the Hi100 diet. This trend may point to Ni’s ability to bioaccumulate in zebrafish, as evidenced for Hg and As too. Several studies have demonstrated Ni accumulation in various fish tissues, including gill, kidney, skeleton, white muscle, liver, brain, heart, stomach, intestine, skin, scales, and gonads, following either waterborne or dietary Ni exposure [29,113].

Ni was the metal present in the highest concentrations in zebrafish, but Ni is not considered in the laws that limit PTEs content in feed and food. Considering the level of this metal detected in zebrafish and taking into account its toxicity, the authors think that it would be interesting to deepen this topic and suggest the scientific community should identify and fix specific limits for Ni content both in feed and food.

**Table 4:** Potentially toxic elements (PTEs) content in zebrafish (*Danio rerio*) laboratory cultured: comparison with literature data.

Life Stage	Organ	Exposure	PTEs Content (mg kg^−1^ ww or dw)	Ref
**Cd**				
larvae	whole	natural	0.044–0.054 (dw) 0.0068–0.0082 (ww)	this study
juvenile	carcass		0.32–0.73 (dw) 0.08–0.16 (ww)	this study
adult	carcass		0.35–0.79 (dw) 0.11–0.25 (ww)	this study
larvae	carcass	natural	~0.02 (ww)	[33]
adult	carcass	natural	0.02 (dw)	[33]
adult	whole	natural	1.5 (dw)	[10]
		10 ppt	~8 (dw)	
adult	brain	natural	0.4 (ww)	[71]
		1 ppm (2 days)	20.2 (ww)	
adult	muscle	natural	0.200 ± 0.03 (ww)	[75]
		1.9 0.6 ppb	0.18 ± 0.04 (ww)	
		9.6 ± 2.9 ppb	0.14 ± 0.04 (ww)	
embryos	whole		~0.2 (dw)	[70]
adult	liver and gills	river water	4–27 (ww)	[68]
adult	carcass	0.5 ppm	~0.75 (ww)	[83]
**Pb**				
larvae	whole	natural	0.16–0.66 (dw) 0.025–0.101 (ww)	this study
juvenile	carcass		0.23–1.19 (dw) 0.055–0.26 (ww)	this study
adult	carcass		0.23–1.30 (dw) 0.072–0.42 (ww)	this study
adult	liver and gills	river water	0.2–38 (ww)	[68]
adult	carcass	1 ppm	~2 (ww)	[83]
**Hg**				
larvae	whole	natural	0.009 ± 0.003 (dw) 0.0013 ± 0.0004 (ww)	this study
juvenile	carcass		0.079 ± 0.003 (dw) 0.018 ± 0.001 (ww)	this study
adult	carcass		0.102 ± 0.005 (dw) 0.032 ± 0.002 (ww)	this study
adult	muscle	natural	next to 0 (dw)	[76]
		5 ppm (7 days)	~3 (dw)	
		5 ppm (21 days)	~8 (dw)	
		5 ppm (63 days)	~15 (dw)	
adult	muscle	60 ± 10 ppb (diet)	~0.6 (dw)	[72]
		11.6 ± 0.4 ppm	~30 (dw)	
**As**				
larvae	whole	natural	0.39–0.52 (dw) 0.060–0.080 (ww)	this study
juvenile	carcass		0.11–0.67 (dw) 0.026–0.16 (ww)	this study
adult	carcass		0.18–0.88 (dw) 0.058–0.27 (ww)	this study
larvae	whole	5 ppm	0.4–0.5 (ww)	[73]
adult	muscle	5 ppm	0.4–0.5 (ww)	
adult		natural	0.94 ± 0.08 (dw)	[77]
adult	carcass	natural	0.27–0.39 (dw)	[33]
adult	liver and gills	river water	4–40 (ww)	[68]
**Ni**				
larvae	whole	natural	0.52–3.3 (dw) 0.089–0.50 (ww)	this study
juvenile	carcass		0.19–4.7 (dw) 0.27–1.03 (ww)	this study
adult	carcass		1.7–6.90 (dw) 0.54–2.1 (ww)	this study
adult	muscle	natural	0.79 ± 0.03 (ww)	[74]
	carcass	natural	2.19 ± 0.19 (ww)	

## 4. Materials and Methods

### 4.1. Ethics

All procedures involving animals were conducted in line with Italian legislation and approved by the Ethics Committee of Università Politecnica delle Marche and the Italian Ministry of Health (626/2018-PR).

### 4.2. Fish Diet Production

Fish diet production was performed according to Zarantoniello et al. [78]. Briefly, full-fat BSF prepupae, reared on a substrate based on coffee silverskin, a coffee industry by-product provided by Saccaria Caffe’ S.R.L. (Marina di Montemarciano, Ancona, Italy), added with 10% (*w*/*w*) of the microalga *Schizochytrium* sp (for details, see [58]), were used to prepare the experimental diets. The Control diet (CTRL) was prepared, according to a commercially available standard diet for zebrafish (Zebrafeed, Sparos Ltd., Olhão, Portugal), with fish meal (FM) and fish oil (FO), wheat gluten, and pea protein concentrates as major ingredients. Insect-based diets were prepared by including different percentage of insect meal (25%, 50%, 75%, and 100%) in substitution of the marine source (FM and FO) in the Control diet (CTRL), to obtain Hi25, Hi50, Hi75, and Hi100 diets, respectively, according to [78].

### 4.3. Experimental Design

Zebrafish specimens were reared as in [78]. Briefly, larvae (collected from embryos) were maintained for 48 h in a Tecniplast system (Varese, Italy) at the following conditions: 28 °C temperature, pH 7.0, NO_2_ and NH_3_ concentrations <0.01 mg/L, NO_3_ concentration <10 mg/L, and a 12L/12D photoperiod. The water in the larval tanks was gently replaced 10 times a day by a dripping system. At 30 days post spawning, fish were gently transferred in big tanks (80 L) equipped with mechanical and biological filtration (Panaque, Capranica, VT, Italy). The duration of the feeding trial was 180 days. Experimental diets were administered starting from 5 to 180 days post fertilization (dpf) twice a day and, in addition, from 5 to 10 dpf, all groups were fed (one feeding in the morning) on the rotifer *Brachionus plicatilis* (5 ind/mL) according to Lawrence et al. [114]. Fish specimens were sampled at 21 (larvae, L), 60 (juveniles, J), and 180 (adults, A) dpf, euthanized with a lethal dose of MS222 (1 g L^−1^), and properly stored for further analyses.

### 4.4. Chemical Analyses and Quality Control

A clean room laboratory ISO 14644–1 Class 6, with areas at ISO Class 5 under laminar flow, was used for sample treatment; all the materials needed to handle samples followed decontamination procedures to avoid any sample contamination, as described in Truzzi et al. [16] and Illuminati et al. [115].

Samples of diets and zebrafish were minced, homogenized (homogenizer MZ 4110, DCG Eltronic, Monza, Italy), and divided into aliquots of 0.5 g each. To determine the moisture, samples were accurately weighed with the analytical balance AT261 (Mettler Toledo, Greifensee, Switzerland) and freeze-dried (Edwards EF4 modulyo, Crawley, Sussex, England) until constant weight (±0.2 mg). Analyses were carried out on three aliquots per sample. For the determination of Cd, Pb, As, and Ni, samples were mineralized in an ultrapure (65% *w*/*v*) nitric acid HNO_3_ and 30% *v*/*v* H_2_O_2_ (Merk, Darmstadt, Germany) mixture in a microwave-accelerated reaction system, MARS-X, 1500 W (CEM, Mathews, NC, USA); operational parameters were as in Truzzi et al. [50]. Agilent DUO 240FS atomic absorption spectrometer (Agilent, Santa Clara, CA 95051, USA) equipped with a graphite furnace (GTA120 Graphite Tube Atomizer) and a Zeeman-effect background correction (GF-AAS) was used for the quantitative determinations of Cd, Pb, Ni, and As. Analytical methodology and instrumental parameters were described in [50]. Total mercury content was quantified by thermal decomposition amalgamation atomic absorption spectrometry using a direct mercury analyzer (DMA-1, Milestone, Sorisole, BG, Italy), according to [116]. The homogenized samples were weighed directly into quartz containers. The optimized reading conditions for mercury determination in fish were the same as for Hg determination in feed and in insects [50]. A calibration curve technique was used for the quantification of mercury content [41].

All analyses were carried out in triplicate. Analytical quality control was achieved using the certified reference material DORM-2 dogfish muscle (National Research Council of Canada). Results (Table 5) were in good agreement with the certified values, and the standard deviation was low, proving the good repeatability of the methods.

Three aliquots of water were collected from each tank to determine the total PTEs. Water samples were diluted with ultrapure grade HCl 2% (Carlo Erba, Milan, Italy) (*v*/*v*) and then Hg, Pb, Cd, and As were determinated with a AFS Titan 8220 spectrofluorometer (Fulltech Instruments, Rome, Italy). Argon 5.0 (99.999% purity) was used as a carrier gas. HCl 2 % (for Cd and Pb determination) or 5% (for Hg and As determination) (*v*/*v*) was used as the sample carrier. Reductant agents to produce metal hydrides were H_4_BNa 0.05% (≥98.0%, Merck, Kenilworth, New Jersey, United States) in NaOH 0.4% (*m*/*v*) (99.99%, metal basis, Thermo Fisher, Kandel, Germany) for Hg determination; H_4_BNa 2.0% in NaOH 0.7% and K_3_Fe(CN)_6_ 1.0% (*m*/*v*) (RPE grade, Carlo Erba, Milan, Italy) for Pb determination; H_4_BNa 4.0% in NaOH 0.4%, CH_4_N_2_S 1.0% (*m*/*v*) (RPE grade, Carlo Erba, Milan, Italy), and CoCl_2_ 1.0 µg mL^−1^ (97% Thermo Fisher, Kandel, Germany) for Cd determination; and H_4_BNa 2% in NaOH 0.5% (*m*/*v*) for As determination. AFS instrumental parameters are reported in Table 6. For As determination, samples were diluted with HCl 10% (*v*/*v*), 1% KI (*m*/*v*) (RPE grade, Carlo Erba, Milan, Italy), and 0.5% ascorbic acid (*m*/*v*) (RPE grade, Carlo Erba, Milan, Italy) and analyzed after 1 h. The levels of metals were quantified with a calibration curve.

For Ni determination, samples were mineralized with HNO_3_ 0.2% (*v*/*v*) for 30 min at 90 °C in decontaminated centrifuge tubes and analyzed on a GF-AAS instrument as for diets and fish. Instrumental LOD and LOQ and reference material checks are reported in Table 7. Mineralized DORM-2 (dogfish muscle), NASS-6 (seawater reference material for trace metals), and SLEW-3 (estuarine water reference material for trace metals) (National Research Council of Canada) were used as certified reference material.

### 4.5. Bioaccumulation Factor

The bioaccumulation factor (BAF) was calculated on a dry weight (dw) basis [49], as the ratio between metal concentration in the organism and metal concentration in the feed provided. A BAF greater than 1 suggests bioaccumulation of the element from the diet into the fish.

### 4.6. Statistical Analysis

Data are expressed as mean ± standard deviation (SD) of the performed replications. After testing the homogeneity of variance with Levene’s test, the normal distribution of data was verified. Therefore, data were subjected to the one-way analysis of variance (ANOVA), followed by the multiple range test [117], to evaluate significant differences among groups at the 95% confidence level. When the ANOVA test gave a *p*-value equal to 0.0000, in the text it was indicated as *p* < 0.0001. All statistical treatments were performed using STATGRAPHICS 19 Centurion [118].

## 5. Conclusions

The substitution of FM and FO with *Hermetia illucens* meal in experimental diets led to a reduction in the content of some PTEs, such as Pb, As, and Ni, with concentrations for all studied PTEs below the legal limit of undesirable substances in animal feed given in [45] (however, there are no limits for Ni). Moreover, this study shows that the PTEs content of zebrafish specimens can be influenced both by the type of diet administered and by the life stage of the animal itself. Zebrafish fed with Hi meal-based diets showed a very low content of potentially toxic elements. The metal present in higher concentration, Ni, deserves particular attention because of its toxicity and the lack of a legal limit for its content both in feed and in food. However, results suggested that the strict control of the presence of contaminants throughout the production chain, from the growth substrate of insects to insects, to fish feed, and, finally, to fish specimens, in view of the possible use of insects in fish feed, could make aquaculture an environmentally sustainable and safe sector. This research demonstrates the possibility of using Hi prepupae raised on substrates based on silverskin (coffee-roasting waste) and 10% microalgae *Schizocytrium* sp. as an aquafeed ingredient, without exposing fish to a chemical risk. Consequently, in perspective, this study paves the way for the use of eco-sustainable insect-based diets in aquaculture for the breeding of edible fish species, after a careful assessment of the chemical risk associated with them, to avoid risks to human health.

## Figures and Tables

**Table 1:** PTEs levels in water tank compared to legal limits [43].

Element	Determined Value (µg L^−1^)	Legal Limit (µg L^−1^)
Hg	<0.0006	1
Pb	<0.030	25
Cd	0.008 ± 0.001	5
As	0.51 ± 0.04	10
Ni	3.3 ± 0.5	20

**Table 3:** Bioaccumulation factor (BAF) of PTEs in larval (L), juvenile (J), and adult (A) specimens of zebrafish, calculated on a dry-weight basis.

Sample *	Cd	Pb	Hg	As	Ni
L_CTRL_	0.10 ± 0.01	0.23 ± 0.2	0.20 ± 0.01	**2.0 ± 0.2**	0.49 ± 0.04
L_Hi25_	0.16 ± 0.01	0.34 ± 0.02	0.31 ± 0.02	**2.8 ± 0.4**	0.9 ± 0.1
L_Hi50_	0.09 ± 0.01	0.55 ± 0.03	0.24 ± 0.01	**2.9 ± 0.2**	**2.3 ± 0.3**
L_Hi75_	0.08 ± 0.01	0.87 ± 0.04	0.20 ± 0.01	**3.4 ± 0.1**	**3.1 ± 0.5**
L_Hi100_	0.11 ± 0.01	**1.9 ± 0.1**	0.15 ± 0.01	**3.2 ± 0.4**	**27 ± 3**
J_CTRL_	0.7 ± 0.1	0.32 ± 0.02	**2.2 ± 0.2**	**2.8 ± 0.3**	**2.2 ± 0.1**
J_Hi25_	1.3 ± 0.1	0.47 ± 0.03	**2.0 ± 0.1**	**2.4 ± 0.3**	**1.9 ± 0.2**
J_Hi50_	0.7 ± 0.1	0.84 ± 0.05	**1.8 ± 0.2**	**1.8 ± 0.2**	**5.2 ± 0.8**
J_Hi75_	**1.3 ± 0.1**	0.98 ± 0.07	**2.1 ± 0.3**	0.8 ± 0.1	**10 ± 2**
J_Hi100_	**1.5 ± 0.1**	**3.4 ± 0.1**	**2.1 ± 0.2**	0.9 ± 0.2	**39 ± 3**
A_CTRL_	0.8 ± 0.1	0.32 ± 0.05	**2.8 ± 0.2**	**3.7 ± 0.6**	**3.5 ± 0.6**
A_Hi25_	1.3 ± 0.2	0.65 ± 0.14	**2.7 ± 0.2**	**2.8 ± 0.6**	**2.3 ± 0.4**
A_Hi50_	0.8 ± 0.1	0.91 ± 0.14	**2.4 ± 0.2**	**2.7 ± 0.6**	**7.4 ± 2.0**
A_Hi75_	**1.3 ± 0.1**	**1.2 ± 0.1**	**2.7 ± 0.2**	**1.4 ± 0.3**	**11 ± 2**
A_Hi100_	**1.5 ± 0.2**	**3.7 ± 0.4**	**2.6 ± 0.3**	**1.4 ± 0.2**	**41 ± 6**

* Fish fed diet including 0% (X_CTRL_), 25%, (X_Hi25_), 50% (X_Hi50_), 75% (X_Hi75_), and 100% (X_Hi100_) insect meal. Values highlighted in bold are higher than 1.

**Table 5:** Accuracy test using certified reference material DORM-2 (dogfish muscle), NRC Canada. Data are expressed in mg kg^−1^.

Element	Analytical Method	Analytical Result (*n* = 9)	Certified Value	Δ (%)
Cd	GF-AAS	0.041 ± 0.005	0.043 ± 0.008	−4.7
Pb	GF-AAS	0.067 ± 0.003	0.065 ± 0.007	+3.1
As	GF-AAS	17.6 ± 0.5	18 ± 1.1	−2.2
Ni	GF-AAS	18.8 ± 0.9	19.4 ± 3.1	−3.1
Hg	DMA-1	4.30 ± 0.2	4.64 ± 0.2	−6.1

**Table 6:** AFS Titan 8220 spectrofluorometer analytical parameters.

Instrumental Parameter	Hg	Pb	Cd	As
Lamp current (mA)	30/0	80/40	60/30	60/30
PMT (Volts)	275	285	280	320
Carrier gas (mL min^−1^)	300	300	500	300
Shield gas (mL min^−1^)	1000	800	800	800
Reading time (s)	15	15	15	15
Delay time (s)	0.5	0.5	0.5	0.5
Blank judgement Value (if)	5	10	10	10
Torch height (mm)	10	8	8	8
IFS Step (s × rpm, a = analyte; c = carrier; r = reading)	(a) 10 × 100 (c) (r) 16 × 120	(a) 10 × 100 (c) (r) 16 × 120	(a) 10 × 100 (c) (r) 16 × 120	(a) 10 × 100 (c) (r) 18 × 120

**Table 7:** Instrumental LOD and LOQ and quality control check (certified reference material, CRM, DORM-2, NASS-6, and SLEW-3 (NRC CNRC, Ottawa, ON, Canada) for water analysis.

Element	Instrument	LOD (µg·L^−1^)	LOQ (µg·L^−1^)	CRM		
				Name	Certified Value (µg L^−1^)	Measured Value (µg L^−1^)
Hg	AFS	0.0006	0.006	DORM-2	4.64 ± 0.26 ^a^	4.84 ± 0.22
Pb	AFS	0.03	3	DORM-2	0.065 ± 0.007 ^a^	0.060 ± 0.008
Cd	AFS	0.0005	0.005	NASS-6	0.0311 ± 0.0019	0.031 ± 0.008
As	AFS	0.01	0.13	SLEW-3	1.36 ± 0.09	1.47 ± 0.27
Ni	GFAAS	0.144	1.440	SLEW-3	1.23 ± 0.07	1220 ± 390

^a^ Accuracy test carried out on mineralized CRM. Value expressed in mg kg^−1^. AFS: AFS Titan 8220 spectrofluorometer. GF-AAS: Agilent DUO 240FS atomic absorption spectrometer equipped with a graphite furnace.

## Sample Availability

Samples of the compounds are not available from the authors.
